# 

**Published:** 1895-10-19

**Authors:** 


**Notices of Births, Deaths, and Marriages** are inserted in this column at a charge of 2s. 6d. for an announcement not exceeding four lines.

**Notice to Advertisers and Subscribers.**

All Advertisements, Orders for Copies of The Hospital and Business Communications should be addressed to **THE MANAGER, (Not to The Editor)**, The Hospital, 428, Strand, London, W.C.

The Cost of Subscription, post free, is as follows:—Per week, 2½d.; per quarter, 2s. 8d.; per half-year, 5s. 3d.; per annum, 10s. 6d. Foreign:—Per Annum, 15s. 2d.; payable, in all cases, in advance.

### NOTICE TO CORRESPONDENTS.

All MS., Letters, Books for Review, and other matters intended for the Editor should be addressed The Editor, The Lodge, Porchester Square, London, W.

The Editor cannot undertake to return rejected MS., even when accompanied by stamped directed envelope.

**"Burdett's Hospital Annual," 1895.**

Sixth year of publication. 950 pp. Scarlet cloth, gilt lettered. Price 5s. A most complete guide to British, Colonial, Indian, and American Hospitals, Dispensaries, Nursing and Convalescent Institutions, and Asylums. A few complete sets of the preceding five issues of the "Annual" may still be obtained on application to the Publishers, The Scientific Press (Limited), 428, Strand, London, W.C.

**NOTICE.—Vol. XVIII. of "The Hospital" (April to September, 1895), in handsome cloth binding, is now on sale at the Office of this Journal. Price 6s. Cases for binding the Numbers contained in this Volume, 1s. 6d. Index to Vol. XVIII., 2½d., post free.**

**The Monthly Part for October is now ready, price 6d.; by post, 8d.**

### SCRAPS AND GLEANINGS.

The total amount raised by the Hospital Saturday collections at Peterborough this year came to £51.

We gather that about 25,000 cases of cholera have been numbered since the outbreak of the present epidemic in Japan.

There are ten medical men members of the House of Parliament. Seven of these are Irishmen, two Scotch, and one English.

A brilliant success in every way was Hospital Saturday in Godalming this year, and the financial result exceeded last year's collection.

The architects for the Wilson block of the Delancey Fever Hospital at Cheltenham are Messrs. Prothero and Phillott, late Middleton, Prothero, and Phillott.

Plans for a new lunatic asylum at Ville-Evrard, in France, are about to be prepared. The asylum is to be especially devoted to the reception of victims to alcoholism.

The General Purposes Committee of the Guildford Joint Hospital Board have submitted for adoption the plans for their addition to the isolation hospital building.

So far, a sum of about £4,280 has been collected towards the building fund of the new infirmary at Helensburgh. An addition of about £600 will set the fund entirely free and out of debt.

The Mile End Guardians are proposing electric lighting in workhouse premises. They have forwarded plans to the Local Government Board for the adoption of this scheme at a cost of £5,000.

The metric system will be introduced in the "British Pharmacopœia" in the edition now being published. This will be printed alongside the system of weights and measures which it supersedes.

A second fête was lately arranged by Messrs. James Pain and Sons, the well known London pyrotechnists, in aid of the funds of the Aberdeen Sick Children's Hospital. The fête was held at the Duthrie Park.

This year the house-to-house collection at Middlesbrough in favour of the funds of the local charities was conducted in a most thorough and systematic manner, which evinced itself in a greatly extended response.

Vaccination is held in great respect in Norway and Sweden. In these countries persons cannot legally be united in holy matrimony without the production of a certificate attesting that they are stamped genuinely with vaccine marks.

From statistics relating to zymotic disease, London shows, itself the healthiest among thirty-three of the large towns of England. In the metropolis the average did not exceed 3·3 per thousand of the population during the year.

The site (comprising four acres of land) for the proposed infectious diseases hospital at Belper, Derbyshire, has been most generously given by Mr. Herbert Strutt, J.P. This land, at a moderate estimate, is said to be worth £500.

A Swiss watchmaker has just invented a watch for the blind. In the middle of each figure is set a small peg; when the hour-hand reaches a given hour the peg drops. The blind can thus tell the time of the hours anyhow, by finding which peg is down and counting back to twelve.

The report of the sixty-seventh annual meeting of the subscribers of Ancoats Hospital, Manchester, shows that in the out-patient departments there was an increase of nearly 12 per cent., and that over 400 more patients had been attended this year than last. The increasing cost of maintenance was a source of anxiety, but the committee were glad to be able to state that over £800 had been collected by one lady interested in the welfare of the institution.

The site for the Memorial Hospital at Crewe has been given by the London and North-Western Railway Company, and the cost of the building has been defrayed practically by gifts of £1,000 each. Mr. F. W. Webb, chief mechanical engineer of the London and North-Western Railway, has intimated to the Crewe Cottage Hospital Committee that he will provide the necessary funds to complete the entire building of the Hospital. This means an additional contribution of about £500, Mr. Webb already having given £1,000.

### THE STUDENT OF MEDICINE.

### APPOINTMENTS.

J. Aspinall, M.R.C.S.Eng., L.S.A., appointed Medical Officer of Health for the Smallthorne Urban Sanitary District. F. C. Bottomley, B.A., M.B., B.C.Cantab, appointed Senior Resident Medical Officer of the Royal Free Hospital, W.C. Mr. A. Boulton, appointed Medical Officer of Health to the Horncastle Rural District Council. Dr. Bowes, appointed Medical Officer for the Sixth District of the East Ashford Union. A. Don, M.B., C.M. Edin., appointed Assistant Surgeon, Dundee Royal Infirmary. W. C. Ellis, L.R.C.P., L.R.C.S.Edin., appointed Medical Officer for the Tollerton District of the Easingwold Union. E. C. Garland, L.R.C.P.Edin., M.R.C.S.Eng., appointed Medical Officer of Health for the Borough of Yeovil. D. M. Greig, M.B., C.M.Edin., F.R.C.S.Edin., appointed Surgeon to the Dundee Royal Infirmary. J. H. R. Griffiths, M.B., C.M.Edin., appointed House Surgeon to the Carmarthenshire Infirmary. G. Halley, M.B., C.M.Edin., appointed Assistant Surgeon to the Dundee Royal Infirmary. J. Howard-Jones, D.Sc.Pub.Health, M.B., C.M.Edin., appointed Medical Officer of Health, Medical Officer to the Port Sanitary Authority, &c., of the Newport (Mon.) Town Council. H. Knight, M.R.C.S., L.R.C.P., appointed House Surgeon at the West London Hospital, Hammersmith, W. C. V. McCormack, L.R.C.P.Lond., M.R.C.S.Eng., appointed Medical Superintendent of the Corporation Hospital, Bootle, Lancashire. A. G. Merson, L.R.C.P., L.R.C.S.Edin., appointed Parochial Medical Officer for Aberdour and District. C. W. Milner, M.R.C.S., L.R.C.P., appointed Assistant Resident Medical Officer to the Nottingham General Dispensary. Dr. Stanley, appointed Medical Officer for the Fifth District of the East Ashford Union. Mr. H. Stanley, appointed Medical Officer for the Sellindge District of the Eltham Union. W. J. Stephens, M.R.C.S., L.R.C.P., appointed Senior Resident Medical Officer to the Nottingham General Dispensary. J. F. Sykes, M.D., D.Sc., L.R.C.P., M.R.C.S., Medical Officer of Health for St. Pancras, appointed Lecturer on Public Health at Guy's Hospital Medical School. C. Templeman, M.D., D.Sc.Edin., appointed Medical Officer of Health for Dundee. J. S. Tew, M.D., D.P.H., appointed Medical Officer of Health for the West Kent Combined Sanitary District. P. M. Thomas, M.D.Cleveland, L.S.A., appointed Medical Officer for the Conwil District of the Carmarthen Union. R. H. Wilkin, M.R.C.S., L.R.C.P., appointed Medical Officer for the Fifth District of the Risbridge Union. G. A. Williamson, M.A., M.B., C.M.Aberd., appointed District Medical Officer in the Island of Cyprus. J. H. Wilson, M.B., C.M. Edin., appointed Medical Officer of Health for the Standish with Langtree Urban Sanitary District and Medical Officer for the Standish District of the Wigan Union. H. Worsley, M.B., C.M. Edin., appointed Medical Officer of Health for the Church Urban Sanitary District.

THE STUDENT OF MEDICINE—*continued.*

### VACANCIES.

#### Resident Medical Officers.

General Hospital, Birmingham.—Assistant House Surgeon. Appointment for six months. No salary, but residence, board, and washing provided. Applications to Howard J. Collins, House Governor, by October 26th.

Nottingham General Dispensary.—Junior Assistant Resident Surgeon. Appointment for six months. Salary at the rate of £120 per annum, with rooms, fire, and attendance. Applications to the Resident Surgeon, Broad Street, Nottingham, by October 22nd.

Oldham Infirmary.—Junior House Surgeon; doubly qualified. Salary £50 per annum, with board and residence. Applications to E. L. Blake, Secretary, by October 22nd.

Royal Free Hospital, Gray's Inn Road, W.C.—Resident Medical Officer (House Physician), doubly qualified. Appointment for six months, but eligible for re-election. Board, residence, and washing provided. No salary. Applications to the Secretary by October 19th.

St. Marylebone General Dispensary, 77, Welbeck Street, Cavendish Square.—Assistant Resident Medical Officer; doubly qualified. Appointment for six months. Salary at the rate of £50 per annum, with furnished apartments, attendance, coal, and light. Applications to the Directors by October 31st.

Stourbridge Dispensary.—House Surgeon and Secretary. Salary £120 per annum, increasing £5 a year to £130, with furnished rooms, coal, gas, and extra allowance of £25 for travelling expenses. Applications to the Honorary Secretary, T. F. Bland, The Firs, Norton, Stourbridge, by October 24th.

Sussex County Hospital, Brighton.—Fourth Resident Medical Officer, doubly qualified, unmarried, and under 30 years of age. Salary not exceeding £30 per annum, with board, washing, and residence in the hospital. Applications to the Secretary by October 23rd.

#### Non-Resident Medical Officers.

Belgrave Hospital for Children, 77 and 79, Gloucester Street, S.W.—Surgeon to Out-patients; must be F.R.C.S.Eng. Applications to the Honorary Secretary by November 2nd.

Borough of Scarborough.—Medical Officer of Health. Salary for the first year £325, for the second £350, and for the third and following year £375. Will be required to act as Public Analyst at a further salary of £25 per annum. Not less than 25 or more than 40 years of age. Applications to John T. Graham, Town Clerk, Town Hall, Scarborough, by October 21st.

East London Hospital for Children and Dispensary for Women, Glamis Road, Shadwell, E.—Assistant Physician to see out-patients. Must be a Fellow or Member of the Royal College of Physicians of London. Applications to Thomas Hayes, Secretary, by October 26th.

Glasgow Maternity Hospital.—Obstetric Physician and Assistant Obstetric Physician. Applications to Arthur Forbes, Secretary, 146, Buchanan Street, Glasgow, by November 8th.

London Hospital, Whitechapel, E.—Medical Electrician; must be qualified and registered under the Medical Act. Applications to G. Q. Roberts, House Governor, by October 19th.

Royal Berkshire Hospital.—Consulting Dentist; must be registered Licentiate in Dental Surgery. Application to the Secretary ten days before the election on November 5th.

Rural Districts of Buntingford, Hadham, Hertford, Stansted, and Ware, and the Urban Districts of Bishops Stortford, Hertford, Hoddesdon, and Ware.—Medical Officer of Health; must devote his whole time to the office. Salary £600 per annum, including travelling and other expenses. Applications to George H. Gisby, Clerk to the Joint Committee, Council Office, Baldock Street, Ware, Herts, by October 21st.

**Account Books for Institutions; Designed in accordance with the Uniform System of Accounts for Hospitals and Institutions.**

By HENRY C. BURDETT.

Descriptive Price List post free on application.

London: The Scientific Press (Limited), 428, Strand, W.C.

Just Published, Second Edition, crown 8vo., 2s. 6d.
NOTES ON MEDICAL NURSING
*From the Lectures given to the Probationers at the London Hospital.*
By the late JAMES ANDERSON, M.D., F.R.C.P., Assistant Physician to the London Hospital. Edited by E. F. LAMPORT.
With Biographical Notice by the late Sir ANDREW CLARK.
"A large amount of valuable information put in a popular form."—*Medical Chronicle.*
London: H. K. LEWIS, 136, Gower Street, W.C.

The Scientific Press (Limited), 428, Strand, London, W.C.

---

Now Ready. With numerous Illustrations, demy 8vo., blue buckram, gilt, 3s. 6d.

### DIET IN SICKNESS AND IN HEALTH.

**By Mrs. ERNEST HART,**

*Formerly Student of the Faculty of Medicine of Paris, and of the London School of Medicine for Women.*

With an Introduction by Sir HENRY THOMPSON, F.R.C.S., M.B. Lond.

A Practical Treatise on Diet both in Sickness and Health, based upon the most recent scientific and medical knowledge; but written in Popular and Comprehensive Language, and accompanied by a Series of Valuable Recipes for the Feeding of Invalids and others.

Sir Henry Thompson, in his Introduction, says:—"I do not hesitate to express my opinion that the present volume forms a handbook to the subject, which will not only interest the dietetic student, but offer him, within its modest compass, a more complete epitome thereof than any work which has yet come under my notice."

London: THE SCIENTIFIC PRESS (LIMITED), 428, STRAND, W.C.

